# Supplementary material for: Fast Human Motion reconstruction from sparse inertial measurement units considering the human shape
Source: Nat Commun. 2024 Mar 18;15:2423. doi: 10.1038/s41467-024-46662-5 (PMC10948800; doi:10.1038/s41467-024-46662-5)
Supplement: Supplementary file 1 — Supplementary Information [file 41467_2024_46662_MOESM1_ESM.pdf]

Supplementary Information

Fast Human Motion reconstruction from sparse inertial measurement units considering the human shape

Xuan Xiao[1,2], Jianjian Wang[1,2], Pingfa Feng[1,2], Ao Gong[1,2], Xiangyu Zhang[1,2], and Jianfu Zhang[1,2,*]

[1]State Key Laboratory of Tribology in Advanced Equipment, Department of Mechanical Engineering, Tsinghua University, Beijing, 100084, China
[2]Beijing Key Lab of Precision/Ultra-precision Manufacturing Equipments and Control, Department of Mechanical Engineering, Tsinghua University, Beijing, 100084, China
[*]zhjf@tsinghua.edu.cn

A Details

A.1 Submodules of the pipeline

This section introduces various submodules of the FIP pipeline to help readers better understand the details. These include the fundamental NN block (Fig. 1), the T-poser regressor (Fig. 2), the shared integrator (Fig. 3), the half body positions regressor (Fig. 4) and the IK solver (Fig. 5).

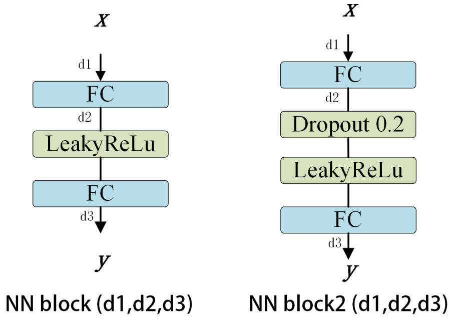

Supplementary Figure 1. Basic NN (Neural Net) blocks of the pipeline. "FC" means fully connected layer.

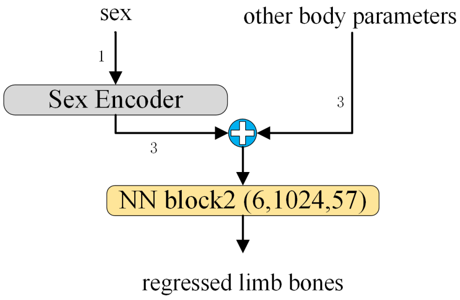

Supplementary Figure 2. Structure of the T-pose regressor. The Sex Encoder uses the approach of word embedding.

A.2 Loss function

For supervising rotation, we used the 6D representation because Zhou et al. demonstrated that this representation yields more accurate results in kinematic inverse solving[1]. The loss term can be calculated with the following Eq. 1:

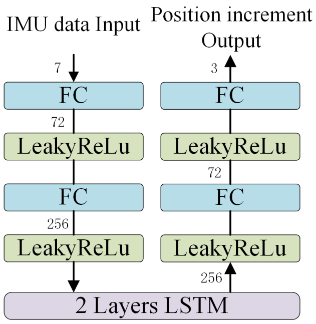

Supplementary Figure 3. Structure of the shared integral regressor. It shares the weights among different sensors.

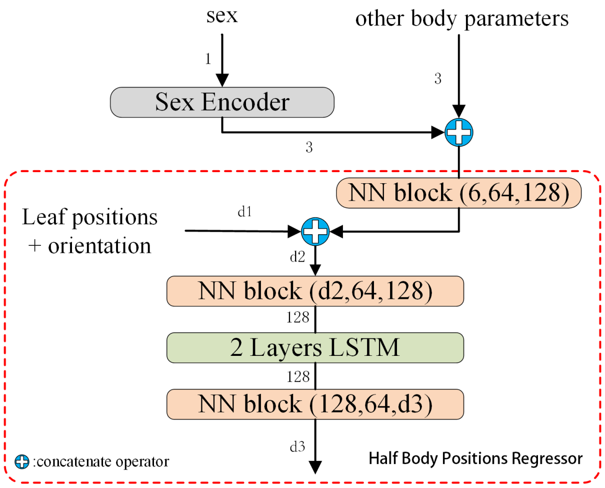

Supplementary Figure 4. Structure of the half body positions regressor. It predicts the full position of the half-body nodes in the SMPL skeleton from the body shape parameters, leaf node positions and sensor signals.

$$L_{rot}(t) = ||\mathbf{R}^{(\mathbf{6D})}_{\mathbf{all,gt}}(\mathbf{t}) - \mathbf{R}^{(\mathbf{6D})}_{\mathbf{all,pred}}(\mathbf{t})||^{\mathbf{2}} \tag{1}$$

For the full-body joint positions output by the joint position regressors, we calculate the MSE loss for supervision with the following Eq. 2:

$$L_j(t) = ||\mathbf{P}_{\mathbf{all,gt}}(\mathbf{t}) - \mathbf{P}_{\mathbf{all,pred}}(\mathbf{t})||^{\mathbf{2}} \tag{2}$$

During training, for the output rotation, due to the high efficiency of our model, we make rotations perform forward kinematic inference again to obtain the reconstructed node positions. This loss (Eq. 3) can be used to supervise the training of the inverse kinematic solver well.

$$L_{re}(t) = ||\mathbf{P}_{\mathbf{all,gt}}(\mathbf{t}) - \mathbf{P}_{\mathbf{all,reconstruct}}(\mathbf{t})||^{\mathbf{2}} \tag{3}$$

In addition to the rotation and position of reconstruction, we supervise the predicted skeleton. The skeleton $S \in \mathbb{R}^{57}$ is composed of 19 limbs in SMPL. During the training process, we supervise the difference between the real skeleton and the predicted skeleton, as shown in Eq. 4.

$$L_{skeleton} = ||\mathbf{S}_{\mathbf{gt}} - \mathbf{S}_{\mathbf{predict}}||^{\mathbf{2}} \tag{4}$$

During training, the weights of later frames are set higher. This is true for all the abovementioned loss functions.

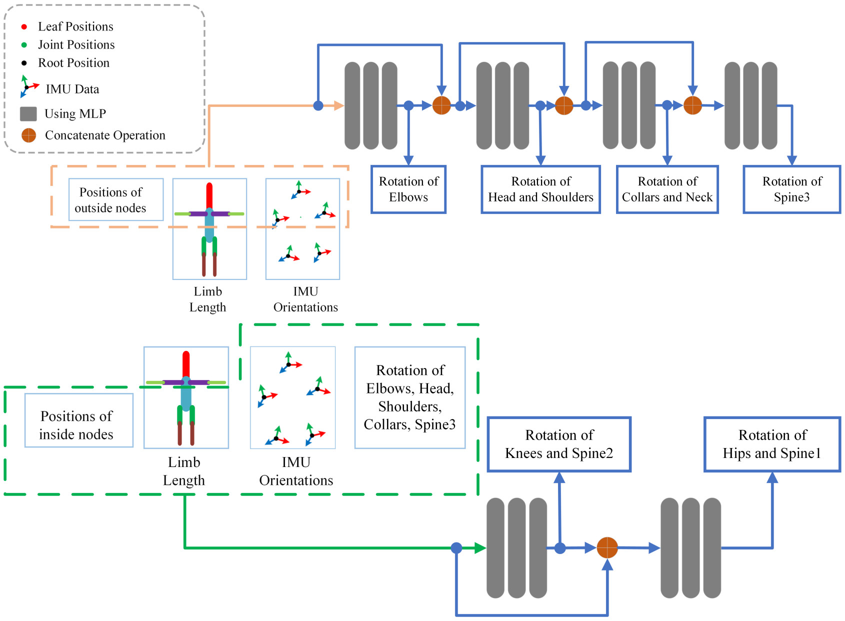

Supplementary Figure 5. Structure of the Inverse Kinematic Solver (IK solver). The full IKsolver consists of two parts with similar structures. They output the nodes' rotations of the kinematic tree in a ladder fashion.

According to the loss function above, the total loss can be written as Eq. 5 and Eq. 6(α and β are the hyperparameters decided artificially):

$$L_{total} = \alpha L_{skeleton} + \sum_t w_t(\alpha(L_{re}(t) + L_j(t)) + \beta L_{rot}(t)) \tag{5}$$

$$w_t = \frac{t}{\sum_0^{length} t} \tag{6}$$

A.3 Dataset

We used the AMASS[2] dataset and the DIP-IMU[3] dataset when training our model. The AMASS dataset provides accurate joint rotations based on the SMPL skeleton, containing 500 subjects, over 3,000 minutes, and over 10,000 actions. The DIP-IMU dataset provides IMU real data (acceleration and orientation) and the joint rotation angle of the SMPL skeleton, including 10 subjects and more than 50 sets of actions. Similar to the methods compared in this paper, the larger AMASS dataset is used for pre-training, and the DIP-IMU dataset is used for fine-tuning.

A.4 Implementation details

IMU Data Normalization. To convert the sensor signal from the world coordinate system to the human body coordinate system, following DIP[3], we perform the normalization process in Eq. 7 and 8. $\overline{\mathbf{R}}_{l,s}(t)$ is the normalized orientation at time step t of the leaf sensor, while $[\mathbf{R}_{root}(t)]^{-1}, \mathbf{R}_{l,s}(t)$ are calibrated data. Similarly, $\bar{\mathbf{a}}_{l,s}(t)$ is the normalized acceleration, while $\mathbf{a}_{root}^{-1}(t), \mathbf{a}_{l,s}(t)$ are calibrated data. Typically, the raw orientation data from IMUs is output in the form of quaternions. However, it needs to be converted into the form of rotation matrices before normalizing IMU data for ease of input into the model.

$$\overline{\mathbf{R}}_{l,s}(t) = [\mathbf{R}_{root}(t)]^{-1} \cdot \mathbf{R}_{l,s}(t) \tag{7}$$

$$\bar{\mathbf{a}}_{l,s}(t) = [\mathbf{R}_{root}(t)]^{-1} \cdot \left(\mathbf{a}_{l,s}(t) - \mathbf{a}_{root}(t)\right) \tag{8}$$

Body Parameter Calculation. In the model input, the user's sex, height, arm length, and leg length are included. The sex and height are common parameters. The arm and leg lengths are measured by T-pose; e.g. for the SMPL model, the arm length can be calculated with Eq. 9, while the leg length can be calculated with Eq. 10.

$$l_{arm} = \frac{|x_{20} - x_{16}| + |x_{21} - x_{17}|}{2} \tag{9}$$

$$l_{leg} = \frac{|y_1 - y_7| + |y_8 - y_2|}{2} \tag{10}$$

In Eq. 9 and 10, x_n is the x coordinate of the nth node, while y_n is the y coordinate, in Tpose of the SMPL coordinate system. To regress the DIP-IMU object body parameters, we calculate the average of the parameters from all objects in the AMASS[2] dataset, whose height error ranges within 5 cm and the sex is the same as the ground truth, based on details provided by the DIP-IMU dataset.

Model training. Similar to other methods, we pretrain the model on the synthesis data from the AMASS dataset and fine-tune the model on the training subset of the DIP-IMU dataset. Finally, it is tested on the test partition of the DIP-IMU dataset. It is worth noting that the data of the first 8 collectors of DIP-IMU were selected for fine-tuning, and the data of another 2 collectors were used for testing.

The synthesis acceleration measurements are calculated with Eq. 11:

$$\mathbf{a}_i(t) = \frac{\mathbf{x}_i(t-3) + \mathbf{x}_i(t+3) - 2\mathbf{x}_i(t)}{(3\Delta t)^2} \tag{11}$$

where $x_i(t)$ is the coordinate of the ith IMU at frame t, and $a_i(t)$ is the synthesis acceleration measurement.

We use a graphics workstation with an Intel(R) Xeon(R) Gold 5218R CPU and an Nvidia Quadro RTX 5000 graphics card to train and evaluate the weights of the neural network. Furthermore, we use an Nvidia Jetson TX2 NX[4]-embedded computer with 4G shared memory and a GPU, which is composed of an Nvidia Pascal architecture with 256 CUDA cores, to test the efficiency of our model on mobile terminals. For the graphics workstation, we use PyTorch 1.8 and CUDA 11.2 for training and evaluation. For the TX2 NX card, we use Jetpack PyTorch 1.8 and CUDA 10.2 for evaluation. A 60 fps sensor input is assumed for both training and evaluation. The training data are additionally clipped into sequences with 200-frame lengths. We train the model with a batch size of 64 and 3k epochs (almost the minimum point of the test error curve) using the Adam[5] optimizer with learning rate of 1×10^{-4}. For visualization, we use the human_body_prior package[6] for Python static visualization and Unity3D[7] for a live demo. We use Noitom[8] sensors for our demo.

A.5 Experimental configuration

It should be noted that during the deployment experiment in this paper, the same environment configuration is used for different methods. TPF was tested on a graphics workstation with an Intel(R) Xeon(R) Gold 5218R CPU and an Nvidia Quadro RTX 5000 graphics card, using PyTorch 1.8 and CUDA 10.2. When testing FPS and latency on an embedded computer, the same Nvidia TX2NX module equipped with Jetpack PyTorch 1.8 and CUDA 10.2 is used. The deployment metrics are worse than those mentioned in the previous papers because Nvidia TX2NX is an embedded computer with low computing power. All methods were provided by their official GitHub (pre-trained on the AMASS dataset and fine-tuned on the DIP-IMU dataset, consistent with the method in this paper).

A.6 Comparation configuration

Regarding the source code of PIP[9], the author pointed out that using the CPU in the inference phase would achieve faster and more real-time results than GPU calculations. According to the analysis, this may because PIP adopts an action optimization library based on CPU calculation. Therefore, the deployment counting performance during evaluation is calculated according to the CPU used as it prompts. The latency calculation is shown as Eq. (12):

$$Latency = AFF \cdot FPS_s + TPF \tag{12}$$

AFF is the required amount of future frame, FPS_s is the frame rate of sensor data, and TPF is the single frame inference time. Among the compared methods, Transpose and TIP use the data of the next 5 frames, and the rest of the methods do not. Thus the latency of Transpose and TIP is high.

A.7 IMU (Inertial Measurement Unit)

The raw measurement of a 9-axis IMU is a 3-axis gyroscope, 3-axis accelerometer, and 3-axis magnetometer. Since the gyroscope and magnetometer data cannot be directly used for motion capture, the IMU can output a more accurate orientation through the internal fusion algorithm. The data the method uses is the filtered orientation (rotation matrix or quaternion) and acceleration.

A.8 Details of the demo

A.8.1 IMU used

The utilized device in the demonstration is the Noitom PN3 device[10], which is capable of transmitting IMU data at a rate of 60 frames per second. With its angular resolution of 0.02°, it ensures high accuracy in capturing orientation changes. The sensor outputs data in the east north up (ENU) coordinate system. During the secondary development of PN3, it can directly output the raw acceleration values and the filtered orientation through internal algorithms. Therefore, prior to utilization, the sensor data requires normalization and alignment with the global coordinate system.

Once the sensors are worn, the human body assumes the Tpose position, serving as the reference for the initial frame calibration of the algorithm. During this calibration process, the human coordinate system coincides with the global coordinate system in the first frame. Consequently, calibration is performed based on the following formula:

$$\mathbf{R}_{g,b}(t) = \mathbf{R}_{s2b}\mathbf{R}_{s,b}(0)^{-1}\mathbf{R}_{s,b}(t)\mathbf{R}_{s2b}^{-1} \tag{13}$$

$$\mathbf{a}_{g,b}(t) = \mathbf{R}_{g,b}(t)\mathbf{a}_{s,b}(t) \tag{14}$$

$\mathbf{R}_{s,b}(t)$ represents the orientation output of the sensor worn at position b at time t. $\mathbf{R}_{s2b}$ denotes the rotation matrix that transforms the sensor's coordinate system to align with the human coordinate system, as determined during the process of sensor placement. Specifically, during the Tpose calibration pose, when the sensor coordinate system aligns with the human coordinate system, the rotation matrix is defined as the identity matrix. $\mathbf{R}_{g,b}(t)$ represents the global coordinate orientation of the sensor worn at position b at time t. $\mathbf{a}_{g,b}(t)$ corresponds to the global acceleration experienced by the sensor located at position b at time t. $\mathbf{a}_{s,b}(t)$ denotes the acceleration output of the sensor worn at position b at time t.

A.8.2 Translation estimation

Although FIP is primarily used to estimate human body posture, it can be used in conjunction with other techniques to estimate the translation of the root. In the demonstration, we showcased a simple approach that utilizes gait calculations to estimate the root position. During the estimation process, we assumed that the person is moving with a single-foot-touching gait, such as forward or side straddle.

Initially, we utilized FIP to estimate the positions of the two ankle joints. Regarding this estimation, the foot associated with the lower ankle is considered to be in contact with the ground.

Next, we employed the gait movement formula to calculate the translation of the root. The formula is presented below:

$$\mathbf{x}_{root}^{(global)} = -\mathbf{R}_{root}^{(global)}\mathbf{x}_{contact}^{(human)} \tag{15}$$

$\mathbf{x}_{root}^{(global)}$ represents the translation of the root in the global coordinate system. $\mathbf{R}_{root}^{(global)}$ represents the orientation of the root sensor in the global coordinate system. $\mathbf{x}_{contact}^{(human)}$ represents the translation of the contact foot relative to the root in the body coordinate system.

A.9 Other details

The human body coordinate system is defined in Fig.1 of the main text. Specifically, it follows the SMPL[11] coordinate system, where in the T-pose position, the x-axis points toward the left-hand direction, the y-axis points upward, and the z-axis points toward the front of the body.

The global coordinate system remains fixed and does not move with the human's motion. In the experiment, the initial position and orientation of the human body coordinate system are calibrated to align with the global coordinate system.

The method requires four additional parameters: the sex, arm length, leg length, and height. During the usage of the method, sex is represented as a binary variable with 0 for male and 1 for female as inputs to the model. The arm length is measured from the wrist to the armpit in the T-pose position, while the leg length is measured from the ankle to the hip. The height is measured as the vertical distance from the foot to the top of the head. The average values of the left and right arms are used for arm length, and the average values of the left and right legs are used for leg length.

In the training and testing data, the SMPL model is used to estimate these parameters. Specifically, in the T-pose position, the arm length is computed as the average of the horizontal distances between Nodes 17 and 21 and between Nodes 20 and 16. The leg length is computed as the average of the vertical distances between Nodes 2 and 8 and between Nodes 1 and 7. Due to the approximate proportionality between arm span and height, and the fact that the SMPL-X[12] model can represent finger joints, the height is estimated as the horizontal distance between the fingertips of the left and right hands in the T-pose position using the same parameters in the SMPL-X model.

B More results

We conducted a comparison with other methods (DIP[3], Transpose[13], PIP[9] and TIP[14]) in terms of the rotation errors for all nodes in the body. The results are summarized in Tab. 1, where the data is presented in the format of mean (+/-) standard deviation. The results indicate that among the 15 nodes, FIP achieved the optimal value in 8 nodes, followed by 5 nodes for PIP and 2 nodes for TIP. It is worth noting that FIP demonstrates certain advantages in the reconstruction of limbs, such as the hip, knee, and elbow. Particularly in terms of angle error at the elbow, FIP exhibits approximately a 37% reduction compared to PIP.

	DIP	Tanspose	PIP	TIP	FIP(Ours)
Lhip	12.91 (+/- 10.25)	10.98 (+/- 9.48)	9.79 (+/- 8.10)	9.42 (+/- 10.34)	**8.91 (+/- 6.11)**
Rhip	12.14 (+/- 10.11)	10.58 (+/- 9.07)	9.27 (+/- 6.86)	8.71 (+/- 9.89)	**8.61 (+/- 5.69)**
Spine1	8.04 (+/- 5.87)	9.29 (+/- 6.69)	**7.50 (+/- 5.02)**	7.78 (+/- 5.55)	7.99 (+/- 5.51)
Lknee	10.23 (+/- 6.35)	3.60 (+/- 2.46)	4.75 (+/- 3.28)	3.85 (+/- 2.57)	**3.29 (+/- 3.30)**
Rknee	9.35 (+/- 6.15)	3.63 (+/- 2.81)	4.82 (+/- 3.58)	3.70 (+/- 3.01)	**3.60 (+/- 3.52)**
Spine2	10.48 (+/- 7.61)	11.88 (+/- 7.32)	**9.39 (+/- 6.25)**	10.17 (+/- 6.51)	10.62 (+/- 7.14)
Spine3	12.58 (+/- 9.38)	13.33 (+/- 8.19)	**10.90 (+/- 7.45)**	12.16 (+/- 7.86)	13.01 (+/- 8.75)
Neck	8.71 (+/- 5.52)	7.27 (+/- 4.59)	7.75 (+/- 5.02)	**7.06 (+/- 4.45)**	7.49 (+/- 4.89)
Lcollar	18.46 (+/- 11.63)	19.61 (+/- 11.43)	**16.36 (+/- 10.78)**	17.70 (+/- 11.49)	18.49 (+/- 11.60)
Rcollar	18.29 (+/- 10.71)	17.46 (+/- 10.06)	16.81 (+/- 11.15)	**16.81 (+/- 10.67)**	17.19 (+/- 10.29)
Head	9.61 (+/- 5.95)	2.88 (+/- 1.95)	4.57 (+/- 3.32)	3.71 (+/- 2.32)	**2.74 (+/- 3.05)**
Lshoulder	23.72 (+/- 15.86)	23.91 (+/- 14.77)	**20.64 (+/- 14.07)**	23.04 (+/- 15.66)	21.66 (+/- 15.15)
Rshouder	25.19 (+/- 15.31)	22.27 (+/- 12.93)	20.65 (+/- 14.69)	20.82 (+/- 15.19)	**18.78 (+/- 13.99)**
Lelbow	26.59 (+/- 19.79)	6.50 (+/- 4.84)	7.33 (+/- 5.46)	8.39 (+/- 6.77)	**4.39 (+/- 4.22)**
Relbow	27.65 (+/- 21.23)	6.12 (+/- 5.06)	7.12 (+/- 5.77)	7.82 (+/- 6.91)	**4.45 (+/- 4.40)**

Bold demotes the best performance value for each method.

Supplementary Table 1. Comparison of the mean value and standard deviation of the angle errors for all nodes.

Subsequently, we conducted a straightforward comparison of accuracy metrics on the TotalCapture dataset. We selected one metric from angle-related errors and one from position-related errors, which include Aang, Pos, and Jitter. Since TotalCapture dataset does not provide any body shape-related parameters, we were only able to set SMPL's shape parameters to 0 to obtain average body shape parameters (corresponding to the "average shape" mentioned in the main text), which reduced the accuracy of FIP.

	Aang (deg)	Pos (cm)	Jitter (km/s^3)
DIP	17.22	9.42	3.62
Tanspose	12.89	6.55	0.95
PIP	**12.04**	**5.61**	**0.20**
TIP	13.55	5.65	0.87
FIP(Ours)	14.47	5.92	1.80

Bold demotes the best performance value for each metric.

Supplementary Table 2. Comparison of the mean value of the errors on TotalCapture dataset.

C Explanations

C.1 The relationship between $\mathbf{R}^{(6D)}$ and $\mathbf{R}$.

Zhou et al.[1] proposed improving the accuracy of pose estimation by supervising training with the continuous 6D representation of rotations. The specific conversion relationship is as follows.

The conversion from the ground truth rotation matrix to the 6D representation is as follows:

$$\mathbf{R_{GT}} = \begin{bmatrix} r_{11} & r_{12} & r_{13} \\ r_{21} & r_{22} & r_{23} \\ r_{31} & r_{32} & r_{33} \end{bmatrix} \tag{16}$$

$$\mathbf{R_{GT}^{(6D)}} = \begin{bmatrix} r_{11} & r_{21} & r_{31} & r_{12} & r_{22} & r_{32} \end{bmatrix}^T \tag{17}$$

The conversion from the predicted 6D representation to the rotation matrix is as follows:

$$\mathbf{R_{pred}^{(6D)}} = \begin{bmatrix} r'_1 & r'_2 & r'_3 & r'_4 & r'_5 & r'_6 \end{bmatrix}^T \tag{18}$$

$$r_{norm}[1] = \| \begin{bmatrix} r'_1 & r'_2 & r'_3 \end{bmatrix}^T \|_2 \tag{19}$$

$$r_{norm}[2] = \| \begin{bmatrix} r'_4 & r'_5 & r'_6 \end{bmatrix}^T \|_2 \tag{20}$$

$$\begin{bmatrix} r'_7 \\ r'_8 \\ r'_9 \end{bmatrix} = \frac{\begin{bmatrix} r'_1 & r'_2 & r'_3 \end{bmatrix}^T}{r_{norm}[1]} \times \frac{\begin{bmatrix} r'_4 & r'_5 & r'_6 \end{bmatrix}^T}{r_{norm}[2]} \tag{21}$$

$$\mathbf{R_{pred}} = \begin{bmatrix} r'_1/r_{norm}[1] & r'_4/r_{norm}[2] & r'_7 \\ r'_2/r_{norm}[1] & r'_5/r_{norm}[2] & r'_8 \\ r'_3/r_{norm}[1] & r'_6/r_{norm}[2] & r'_9 \end{bmatrix} \tag{22}$$

The reason for the additional calculations in equations 8 and 9 when converting the predicted 6D representation to a rotation matrix is that the neural network's output cannot guarantee to be unit vectors.

C.2 Explanations of the network structure

In the neural network design, we adopted a shared integration regressor to estimate the displacements of different sensors. This helps reduce the parameter count of the displacement regressor and is more consistent with the physical laws (the integration process should be the same for different sensors).

Additionally, our method estimates the upper and lower body separately at the node level. First, this reduces the parameter count of individual regressors, resulting in faster inference speed. Second, it mitigates the influence of the lower body on the overall estimation to some extent (as shown in Tab. 1, FIP provides more accurate estimates for more joint angles). However, the coupling between the upper and lower body movements cannot be completely decoupled by independent regression models. This is because, first, during the estimation of node positions, the estimated skeleton is divided into upper and lower body inputs, which are generated by a neural network, thus introducing a certain level of coupling. Second, this method is data-driven, and during the training process, there may exist "latent" connections between the parameters of the two independent regressors. Finally, the method does not fully decouple the estimation of the upper and lower body, since they are still unified in the kinematic inverse process.

C.3 Analysis of deployment improvement

As revealed in the result comparisons presented in the main text, FIP exhibits significant improvements in computational efficiency and deployment performance while maintaining reconstruction accuracy compared to other methods. Through analysis, these improvements primarily stem from the following aspects:

1. Without incorporating any additional optimization designs: It is worth noting that other methods, such as PIP and TIP, employ optimization designs to achieve better reconstruction results. These methods construct optimization problems to improve the reliability of the reconstruction results. For instance, PIP constructs torque constraints to optimize the output of the Transpose algorithm. However, the optimization process often involves multiple iterations to obtain the optimal solution, which is why these methods can be slower during runtime.

2. Efficient expressive power of the network architecture: Since FIP does not employ additional optimization designs, it places a greater emphasis on the expressive power of the model. First, the model incorporates human body parameters to estimate the skeletal structure, providing implicit constraints for the node position estimation module and the inverse kinematics solver. Second, FIP utilizes a shared network to estimate the position increments of different sensors, which aligns with the objective physical observation process. Third, FIP constructs an inverse kinematics solver based on the joint motion tree of the human body, which enhances the expressive capacity of the network to a certain extent, as evidenced by the ablation experiment presented in the main text.

3. Removal of bidirectional propagation design in RNN: In contrast to DIP and Transpose, FIP achieves motion reconstruction using a unidirectional propagation RNN. Bidirectional propagation in RNN not only requires additional future frame

data but also involves simultaneous computation of hidden layer states for multiple future frames, which increases latency and reduces computational speed. However, in their respective papers (DIP and Transpose), it is discussed that switching from bidirectional to unidirectional propagation may result in decreased reconstruction accuracy. PIP, based on the unidirectional propagation results of Transpose, incorporates optimization designs to effectively enhance reconstruction accuracy. On the other hand, FIP benefits from its well-designed network architecture, which not only eliminates the need for result optimization but also removes the bidirectional propagation design in RNN, while still achieving results comparable to PIP. This key advantage enables FIP to enhance computational efficiency without reducing reconstruction accuracy.

References

1. Zhou, Y., Barnes, C., Lu, J., Yang, J. & Li, H. On the Continuity of Rotation Representations in Neural Networks. In *2019 IEEE/CVF Conference on Computer Vision and Pattern Recognition (CVPR)*, 5738–5746, DOI: 10.1109/CVPR.2019.00589 (IEEE, Long Beach, CA, USA, 2019).

2. Mahmood, N., Ghorbani, N., Troje, N. F., Pons-Moll, G. & Black, M. J. AMASS: Archive of motion capture as surface shapes. In *International Conference on Computer Vision*, 5442–5451 (2019).

3. Huang, Y. *et al.* Deep inertial poser: learning to reconstruct human pose from sparse inertial measurements in real time. *ACM Trans. Graph.* **37**, DOI: 10.1145/3272127.3275108 (2018).

4. Nvidia. Jetson tx2 nx module. Website. https://www.nvidia.com/en-us/autonomous-machines/embedded-systems/jetson-tx2/.

5. Kingma, D. P. & Ba, J. Adam: A method for stochastic optimization. *CoRR* **abs/1412.6980** (2014).

6. nghorbani. human body prior. Website. https://github.com/nghorbani/human_body_prior.

7. Unity. The world's leading platform for real-time content creation. Website. https://unity.com.

8. Noitom. Perception neuron series. Website. https://www.noitom.com/.

9. Yi, X. *et al.* Physical Inertial Poser (PIP): Physics-aware Real-time Human Motion Tracking from Sparse Inertial Sensors. In *2022 IEEE/CVF Conference on Computer Vision and Pattern Recognition (CVPR)*, 13157–13168, DOI: 10.1109/CVPR52688.2022.01282 (IEEE, New Orleans, LA, USA, 2022).

10. Noitom. Perception neuron series. Website. https://neuronmocap.com/pages/perception-neuron-3/.

11. Loper, M., Mahmood, N., Romero, J., Pons-Moll, G. & Black, M. J. SMPL: a skinned multi-person linear model. *ACM Transactions on Graph.* **34**, 1–16, DOI: 10.1145/2816795.2818013 (2015).

12. Pavlakos, G. *et al.* Expressive body capture: 3d hands, face, and body from a single image. In *Proceedings IEEE Conf. on Computer Vision and Pattern Recognition (CVPR)* (2019).

13. Yi, X., Zhou, Y. & Xu, F. Transpose: Real-time 3d human translation and pose estimation with six inertial sensors. *ACM Transactions on Graph.* **40** (2021).

14. Jiang, Y. *et al.* Transformer inertial poser: Real-time human motion reconstruction from sparse imus with simultaneous terrain generation. In *SIGGRAPH Asia 2022 Conference Papers*, SA '22 Conference Papers, DOI: 10.1145/3550469.3555428 (2022).
