# Supplementary material for: Fast Human Motion reconstruction from sparse inertial measurement units considering the human shape
Source: Nat Commun. 2024 Mar 18;15:2423. doi: 10.1038/s41467-024-46662-5 (PMC10948800; doi:10.1038/s41467-024-46662-5)
Supplement: Supplementary file 3 — Description of Additional Supplementary Files [file 41467_2024_46662_MOESM3_ESM.pdf]

Description of Additional Supplementary Files

File Name: Supplementary Movie 1
Description: Live demo of the Fast Inertial Poser
