# Supplementary material for: Fast Human Motion reconstruction from sparse inertial measurement units considering the human shape
Source: Nat Commun. 2024 Mar 18;15:2423. doi: 10.1038/s41467-024-46662-5 (PMC10948800; doi:10.1038/s41467-024-46662-5)
Supplement: Supplementary file 5 — Reporting Summary [file 41467_2024_46662_MOESM5_ESM.pdf]

nature portfolio

Corresponding author(s): Jianfu Zhang

Last updated by author(s): 2024/2/24

Reporting Summary

Nature Portfolio wishes to improve the reproducibility of the work that we publish. This form provides structure for consistency and transparency in reporting. For further information on Nature Portfolio policies, see our Editorial Policies and the Editorial Policy Checklist.

Please do not complete any field with "not applicable" or n/a. Refer to the help text for what text to use if an item is not relevant to your study.
For final submission: please carefully check your responses for accuracy; you will not be able to make changes later.

Statistics

For all statistical analyses, confirm that the following items are present in the figure legend, table legend, main text, or Methods section.

n/a	Confirmed	
☐	√	The exact sample size (*n*) for each experimental group/condition, given as a discrete number and unit of measurement
☐	√	A statement on whether measurements were taken from distinct samples or whether the same sample was measured repeatedly
☐	√	The statistical test(s) used AND whether they are one- or two-sided<br>*Only common tests should be described solely by name; describe more complex techniques in the Methods section.*
☐	√	A description of all covariates tested
☐	√	A description of any assumptions or corrections, such as tests of normality and adjustment for multiple comparisons
☐	√	A full description of the statistical parameters including central tendency (e.g. means) or other basic estimates (e.g. regression coefficient) AND variation (e.g. standard deviation) or associated estimates of uncertainty (e.g. confidence intervals)
☐	√	For null hypothesis testing, the test statistic (e.g. *F*, *t*, *r*) with confidence intervals, effect sizes, degrees of freedom and *P* value noted<br>*Give P values as exact values whenever suitable.*
☐	√	For Bayesian analysis, information on the choice of priors and Markov chain Monte Carlo settings
☐	√	For hierarchical and complex designs, identification of the appropriate level for tests and full reporting of outcomes
☐	√	Estimates of effect sizes (e.g. Cohen's *d*, Pearson's *r*), indicating how they were calculated

Our web collection on statistics for biologists contains articles on many of the points above.

Software and code

Policy information about availability of computer code

Data collection	Public dataset AMASS dataset, including: ACCAD, ACCADrenders, BMLhandball, BMLmovi, BMLrub, CMU, DanceDB, DFauset67, EKUT, EyesJapanDataset, KIT, MPIHDM05, MPLILimists, TotalCapture. Public dataset DIP-IMU. Noitom perception neuron 3 IMU device.
Data analysis	Pycharm (version: Professor 2022.1.1). Python package: PyTorch (version:1.8), matplotlib(version: 3.7.2), numpy(version:1.23.5), human_body_prior(v2.2.2.0), jupyterlab (version: 4.0.4). Axis Studio. Unity 2021.3.13f1c1. Clion(version:22.2). C++ library: MocapApi

For manuscripts utilizing custom algorithms or software that are central to the research but not yet described in published literature, software must be made available to editors and reviewers. We strongly encourage code deposition in a community repository (e.g. GitHub). See the Nature Portfolio guidelines for submitting code & software for further information.

Data

Policy information about availability of data

All manuscripts must include a data availability statement. This statement should provide the following information, where applicable:

- Accession codes, unique identifiers, or web links for publicly available datasets
- A description of any restrictions on data availability
- For clinical datasets or third party data, please ensure that the statement adheres to our policy

Any data generated or analyzed during this study, associated protocols, and materials are available from the corresponding author on reasonable request. The data processing and test code, model checkpoint of FIP have been uploaded to the Github repository (github link: https://github.com/bachongyou/FIP_inference)

Research involving human participants, their data, or biological material

Policy information about studies with human participants or human data. See also policy information about sex, gender (identity/presentation), and sexual orientation and race, ethnicity and racism.

Reporting on sex and gender	two males
Reporting on race, ethnicity, or other socially relevant groupings	Asian
Population characteristics	one man of 178 cm height, 70kg; one man of 186 cm height, 75 kg.
Recruitment	Students
Ethics oversight	This work was approved by the Science and Technology Ethics Committee, Tsinghua University

Note that full information on the approval of the study protocol must also be provided in the manuscript.

This checklist template is licensed under a Creative Commons Attribution 4.0 International License, which permits use, sharing, adaptation, distribution and reproduction in any medium or format, as long as you give appropriate credit to the original author(s) and the source, provide a link to the Creative Commons license, and indicate if changes were made. The images or other third party material in this article are included in the article's Creative Commons license, unless indicated otherwise in a credit line to the material. If material is not included in the article's Creative Commons license and your intended use is not permitted by statutory regulation or exceeds the permitted use, you will need to obtain permission directly from the copyright holder. To view a copy of this license, visit http://creativecommons.org/licenses/by/4.0/
